# Agranulocytosis and secondary infection related to JAK inhibitors and IL-6 receptor blockers: a disproportionality analysis using the US Food and drug administration adverse event reporting system

**DOI:** 10.3389/fphar.2023.1323240

**Published:** 2024-01-09

**Authors:** Chunyan Wei, Wanhong Yin, Tingting Hu, Jingyi Zhang, Huifang Dan, Bin Wu

**Affiliations:** ^1^ Department of Pharmacy, West China Hospital, Sichuan University, Chengdu, China; ^2^ Department of Critical Care Medicine, West China Hospital, Sichuan University, Chengdu, China; ^3^ West China School of Clinical Medical College, Sichuan University, Chengdu, China

**Keywords:** janusjanus kinase inhibitors, interleukin-6 receptor blockers, COVID-19, agranulocytosis, secondary infection, FAERS

## Abstract

**Background:** Given that the fight against coronavirus disease 2019 (COVID-19) is not over, we aimed to explore the occurrence of agranulocytosis and infectious complications in patients with and without COVID-19 following immunoregulatory therapy based on real-world data.

**Methods:** This was a retrospective disproportionality analysis based on the US Food and Drug Administration Adverse Event Reporting System (FAERS). All cases reported between the first quarter of 2004 and the fourth quarter of 2022 about Janus kinase inhibitors (baricitinib, tofacitinib, ruxolitinib) and interleukin-6 receptor blockers (tocilizumab, sarilumab) were collected. Disproportionality analyses were conducted by reporting odds ratio (ROR) and information component (IC).

**Results:** A total of 211,363 cases were recognized from the FDA Adverse Event Reporting System database. Data analysis showed that tocilizumab (reporting odds ratio: 3.18, 95% CI: 3.18–3.29; information component: 1.37, 95% CI: 1.31–1.42), sarilumab (ROR: 1.64, 95% CI: 1.55–1.73; IC: 0.61, 95% CI: 0.43–0.79), baricitinib (ROR: 3.42, 95% CI: 3.19–3.67; IC: 1.43, 95% CI: 1.21–1.65), tofacitinib (ROR: 2.53, 95% CI: 2.49–2.57; IC: 1.11, 95% CI: 1.05–1.16), and ruxolitinib (ROR: 1.87, 95% CI: 1.83–1.91; IC: 0.77, 95% CI: 0.70–0.84) were all associated with secondary infection. The association in the combination group was higher than that in the monotherapy group (ROR: 4.69, 95% CI: 4.53–4.86; IC: 1.73, 95% CI: 1.62–1.84). As for agranulocytosis, tocilizumab (ROR: 1.61, 95% CI: 1.53–1.69; IC: 0.67, 95% CI: 0.50–0.84) and ruxolitinib (ROR: 2.32, 95% CI: 2.21–2.43; IC: 1.18, 95% CI: 1.02–1.33) showed the significant signals. The association was higher in the combination group than in the monotherapy group (ROR: 2.36, 95% CI: 2.15–2.58; IC: 1.20, 95% CI: 0.90–1.51). Secondary infection after treatment with tofacitinib (ROR: 1.37, 95% CI: 1.02–1.84), tocilizumab (ROR: 1.46, 95% CI: 1.01–2.09), and sarilumab (ROR: 2.46, 95% CI: 1.10–5.50) was reported more frequently in COVID-19 than in non–COVID-19 patients.

**Conclusion:** Both Janus kinase inhibitors and interleukin-6 receptor blockers are significantly associated with secondary infection and agranulocytosis, and the combined treatment further increases the association. The correlation with secondary infection in patients treated with tofacitinib, tocilizumab, and sarilumab is higher in COVID-19 than in non–COVID-19 patients.

## 1 Introduction

Since the end of 2019, when the coronavirus disease 2019 (COVID-19) pandemic began, more than 3 billion people worldwide have been estimated to have been infected with COVID-19 (COVID-19 Cumulative Infection Collaborators, 2022), and more than 10 million patients died because of COVID-19 (COVID-19 Excess Mortality Collaborators, 2022). On 5 May 2023, the World Health Organization (WHO) declared that COVID-19 no longer constitutes a public health emergency of international concern (PHEIC). However, COVID-19 has not disappeared. Over the past 4 years, clinicians have accumulated extensive experience in treating COVID-19, including antiviral therapy, immunomodulatory therapy, and other supportive treatments (Ahn et al., 2020; Arif et al., 2021; Bilge et al., 2023; Yuan et al., 2023).

Interleukin-6 (IL-6) receptor blockers and Janus kinase (JAK) inhibitors are the most important immunotherapies for COVID-19 (Angriman et al., 2021; Elahi et al., 2022; Levy et al., 2022; RECOVERY Collaborative Group, 2022). IL-6 is one of the most important proinflammatory cytokines, and its increased levels have been recorded in COVID-19 patients, especially in those with severe to critical disease (Coomes and Haghbayan, 2020). IL-6 receptor blockers (such as tocilizumab) are recombinant humanized IgG1 monoclonal antibodies (mAb) that bind to both soluble and membrane-bound receptors of IL-6, thereby inhibiting the IL-6 signaling pathway and reducing the proinflammatory effect of IL-6 (Drożdżal et al., 2021). Therefore, IL-6 receptor blockers have been repurposed to blunt the abnormal COVID-19–related cytokine release. There is increasing evidence for the relevance of IL-6 as a prognostic marker for COVID-19 (Potere et al., 2021). JAK mediates signaling for cytokines and growth factors involved in inflammation, hematopoiesis, and immune response (Drożdżal et al., 2021). JAK inhibitors regulate intracellular signaling by partially inhibiting JAK enzyme activity, thereby reducing the phosphorylation and activation of STAT proteins (Drożdżal et al., 2021). COVID-19–related cytokines act via a family of more than 40 transmembrane receptors that are coupled to one or several of the JAKs coded by the human genome (Levy et al., 2022). JAK inhibitors hold the potential to cut off pathological reactions in COVID-19. For the above reasons, there is already some favorable clinical evidence for the effectiveness of IL-6 receptor blockers and JAK inhibitors in treating COVID-19 (Guimarães et al., 2021; Somers et al., 2021; RECOVERY Collaborative Group, 2022). Therefore, the US Food and Drug Administration (FDA) has approved tocilizumab (IL-6 receptor blocker) and baricitinib (JAK inhibitor) for the treatment of COVID-19. Other JAK inhibitors (e.g., upadacitinib and filgotinib) and interleukin inhibitors lack sufficient evidence of benefit for COVID-19 patients (Drożdżal et al., 2021).

IL-6 receptor blockers and JAK inhibitors were originally developed for the treatment of immune-related diseases, such as rheumatoid arthritis (Gabay et al., 2013; Taylor et al., 2017). It has also been shown that tocilizumab can be used in the treatment of skin diseases (e.g., scleroderma and systemic sclerosis), giant-cell arteritis, and systemic sclerosis–related interstitial lung disease (Stone et al., 2017; Khanna et al., 2022; Mastorino et al., 2022). The clinical application of IL-6 receptor blockers and JAK inhibitors is usually accompanied by some practical problems, including the occurrence of adverse drug reactions (ADRs), such as gastrointestinal reactions and liver function abnormalities. Early studies have shown that these drugs may cause severe ADRs such as agranulocytosis and serious infections (Shreberk-Hassidim et al., 2017; Taylor et al., 2022). The occurrence of agranulocytosis is also one of the risk factors for infectious complications. The FDA has placed black box warnings about the risk of serious infections with the application of JAK inhibitors and IL-6 receptor blockers (Kragstrup et al., 2022; Samuel et al., 2023). The serious ADRs in COVID-19 patients have also begun to attract the attention of researchers. Retrospective studies have shown that the incidence of secondary infection in patients treated with tocilizumab and baricitinib is 32% and 22%, respectively (Peterson et al., 2023). The occurrence of secondary infections may predict a poor prognosis in patients with COVID-19.

The purpose of this retrospective, observational pharmacovigilance study was to analyze the occurrence of agranulocytosis and secondary infections in COVID-19 and non–COVID-19 patients, in monotherapy and combination treatment with IL-6 receptor blockers and JAK inhibitors, based on the FDA Adverse Event Reporting System (FAERS) database, so as to provide a reference for clinicians in balancing the benefits and risks of IL-6 receptor blockers and JAK inhibitors treatment. To the best of our knowledge, this was the first research to analyze the association of IL-6 receptor blockers and JAK inhibitors in monotherapy and combination therapy with secondary infection and agranulocytosis based on the FAERS database.

## 2 Material and methods

### 2.1 Data source

We collected the reports in the FAERS database from the first quarter of 2004 (inception of the database) to the fourth quarter of 2022. The data sources of the FAERS are public and self-reported for post-marketing drug safety monitoring (Wei et al., 2023). ADRs were reported with anonymous patient information. Therefore, no ethical approval was required, and informed consent could not be obtained. We divided patients into the COVID-19 group and the non-COVID-19 group, with non–COVID-19 patients being defined as all patients except those with COVID-19.

### 2.2 Definitive drug inclusion

According to the WHO guideline, two IL-6 receptor blockers (tocilizumab and sarilumab) and three JAK inhibitors (baricitinib, ruxolitinib, and tofacitinib) are recommended for the treatment of COVID-19 (Lamontagne et al., 2020). We identified these five drugs as target drugs. However, only tocilizumab and baricitinib are FDA-approved for the treatment of COVID-19 (Drożdżal et al., 2020; Jain et al., 2023).

### 2.3 Date extraction and processing

The FAERS database references the standardization and classification of ADRs in the Medical Dictionary for Regulatory Activities (MedDRA) (Wei et al., 2023). Each report is coded by preferred terms (PTs) from the MedDRA terminology. One PT may correspond to one or more High-level Terms (HLTs), High-level Group Terms (HLGTs), and System Organ Class (SOC) levels. Through standardized MedDRA queries (SMQs), different PTs can be collected to define specific ADRs (Wei et al., 2023). The FAERS database contains seven modules corresponding to seven aspects of the main content, including patient demographic and administrative information (DEMO), report source (RPSR), drug information (DRUG), adverse events (REAC), patient outcomes (OUTC), indications for drug administration (INDI), and therapy start states and end dates for the reported drugs (THER).

In accordance with the FDA recommendations, a drug-mapped and de-duplicated version of the FAERS data source was extracted (Wu et al., 2022). The general process is described below. First, if the CASEIDs (numbers used to identify FAERS cases) were the same, we selected the latest FDA_DT (date FDA received the case). If the CASEID and FDA_DT were the same, we selected the higher PRIMARYID (a unique number for identifying a FAERS report). Second, MedEx1.3.8 software was used to standardize different names of the same drug into the “generic name” (Wu et al., 2021). Both REAC and INDI modules were coded by MedDRA PTs (Wu et al., 2019).

We collected all the cases of the five drugs (tocilizumab, sarilumab, baricitinib, ruxolitinib, and tofacitinib) as suspected drugs reported in the FAERS database. We divided the reports into three groups, namely, the agranulocytosis group, secondary infection group, and non-target events group. The agranulocytosis and secondary infection events were defined according to the High-Level Group Term, and 2,105 PTs (2084 PTs for infection and 21 PTs for agranulocytosis) were incorporated as shown in Supplementary Table S1. We identified the five drugs in accordance with the WHO Anatomical Therapeutic Chemical (ATC) classification. The ATC codes are shown in Supplementary Table S2. Cases of agranulocytosis and infection reported in the INDI module were excluded.

### 2.4 Statistical analysis

The data source extracted from the FAERS database was managed through Microsoft SQL Server 2017 software in local. We collected the characteristics of cases with target drugs, including age, sex, report country, report year, and identity of the reporters (health professionals or non-health professionals). The reporting odds ratio (ROR) and information component (IC) were used to detect the signals of target events and target drugs (Li et al., 2008). The ROR method was used as the primary assay; if the case number was ≥3 and the lower limit of the 95% confidence interval (95% CI) was >1, the signals were considered significant. The IC value was used as a confirmation method; the significant signals were detected when the IC value was >0 and the lower limit of 95% CI was >0. When both the ROR and IC methods met their threshold, the target events were considered meaningful. We used EXCEL software (version 2304 build 16.0.16327.20200) to calculate the values of ROR, IC, and their 95% CI. Supplementary Table S3 lists the calculation methods. In addition, we used ROR to analyze the differences in reporting frequency of agranulocytosis and secondary infection with the target drugs in COVID-19 and non-COVID-19 patients. The definition rules of signals were the same as above.

## 3 Results

### 3.1 Target-event identification in the FAERS database

**FIGURE 1:**
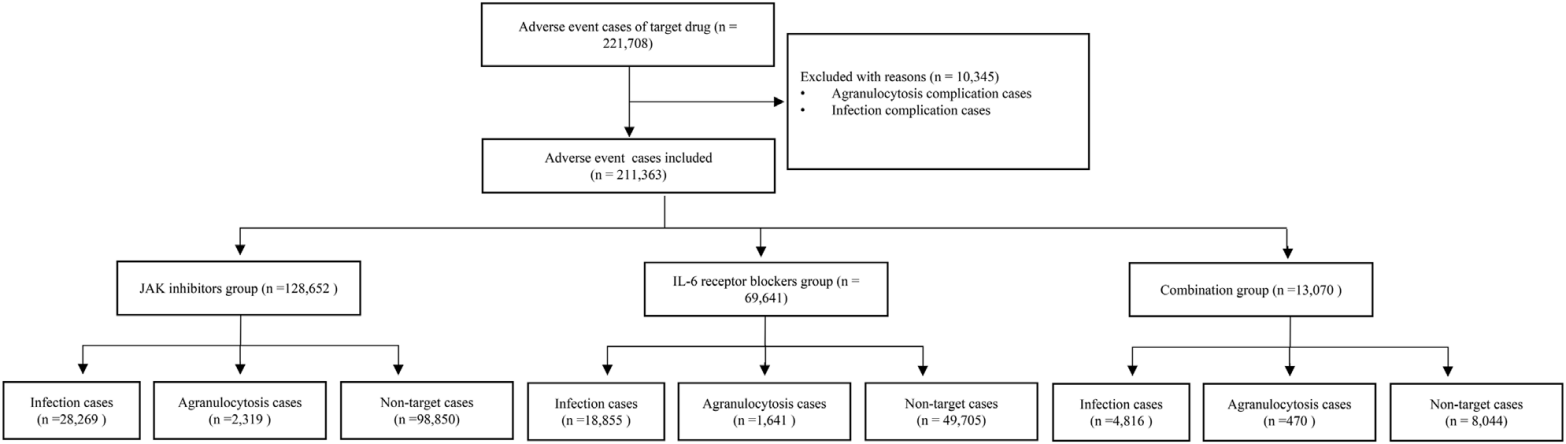
Flowchart of identifying adverse-event cases of target drugs from the FAERS database.

After removing duplicate cases, a total of 221,708 adverse-event cases with the target drugs in the FAERS database were identified, of which 10,345 cases with the complications of infection and agranulocytosis were excluded. Finally, 211,363 cases were included in the disproportionality analysis. A total of 51,940 (24.57%) cases were assigned to the infection group, and 4 430 (2.10%) cases were assigned to the agranulocytosis group. There were 128,652 cases in the JAK inhibitors group, 69,641 cases in the IL-6 receptor blockers group, and 13,070 cases in the combination group. In 1 606 cases, both secondary infection and agranulocytosis were reported. Figure 1 shows the details of the case identification process.

### 3.2 Demographic characteristics

The characteristics of the events are described in Table 1. Both in the infection group and in the agranulocytosis group, the proportion of female was significantly higher. This might be due to the significantly higher incidence of rheumatoid arthritis in females (Myasoedova et al., 2010). However, we separately analyzed the gender characteristics of COVID-19 patients who received JAK inhibitors and IL-6 receptor blockers; regardless of infection group, agranulocytosis group, and non-target events group, the number of female patients reported was significantly higher than that of male patients. This might suggest that the use of JAK inhibitors and IL-6 receptor blockers in female patients with COVID-19 had a higher association with ADRs. Both infection and agranulocytosis were more commonly reported in adults, and were reported more often by health professionals. The United States and Canada were the countries with the highest number of reports, accounting for more than 50% of the total. The number of reports increased year by year, with more than 60% occurring after 2019.

**TABLE 1:** Demographic characteristics of cases.

Category	Infection group		Agranulocytosis group		Non-target events group	
	Number (n)	Proportion (%)	Number (n)	Proportion (%)	Number (n)	Proportion (%)
Gender in total						
Female	33,029	63.59	1,995	45.03	88,966	56.81
Male	9,288	17.88	1,231	27.79	29,265	18.69
Unknown	9,623	18.53	1,204	27.18	38,368	24.50
Gender in COVID-19^a^						
Female	102	69.86	7	70.00	181	61.99
Male	38	26.03	3	30.00	91	31.16
Unknown	6	4.11	0	0.00	20	6.85
Age (years)						
<18	1,179	2.27	543	12.26	2,765	1.77
18–65	19,651	37.83	1,114	25.15	47,763	30.50
≥65	12,483	24.03	919	20.74	31,076	19.84
Unknown	18,627	35.86	1,854	41.85	74,995	47.89
Identity of reporter						
Health professional	21,886	42.14	2,788	62.93	64,125	40.95
Non-health professional	18,420	35.46	791	17.86	71,116	45.41
Unknown	11,634	22.40	851	19.21	21,358	13.64
Occurrence country						
United States	21,460	41.32	1,373	30.99	94,690	60.47
Canada	15,458	29.76	992	22.39	24,637	15.73
Japan	2,242	4.31	344	7.77	5,226	3.34
Germany	2,017	3.88	217	4.90	3,622	2.31
United Kingdom	946	1.82	94	2.12	2,234	1.43
France	816	1.57	183	4.13	2,249	1.44
Other	5,796	11.16	972	21.94	14,565	9.30
Unknown	3,205	6.17	255	5.76	9,376	5.99
Report year						
2004–2013	2,108	4.05	233	5.26	6,706	4.28
2014–2018	16,421	31.62	1,401	31.63	54,401	34.74
2019–2022	33,411	64.33	2,796	63.11	95,492	60.98

^a^ COVID-19: coronavirus disease 2019.

### 3.3 Infection-signal detection

As for IL-6 receptor blockers, both tocilizumab (ROR: 3.18, 95% CI: 3.18–3.29; IC: 1.37, 95% CI: 1.31–1.42) and sarilumab (ROR: 1.64, 95% CI: 1.55–1.73; IC: 0.61, 95% CI: 0.43–0.79) showed the association with secondary infection, and the signal of tocilizumab was higher. As for JAK inhibitors, baricitinib (ROR: 3.42, 95% CI: 3.19–3.67; IC: 1.43, 95% CI: 1.21–1.65), tofacitinib (ROR: 2.53, 95% CI: 2.49–2.57; IC: 1.11, 95% CI: 1.05–1.16), and ruxolitinib (ROR: 1.87, 95% CI: 1.83–1.91; IC: 0.77, 95% CI: 0.70–0.84) were all associated with secondary infection, and the signal of baricitinib was the highest. In the combination group, the signals from the tocilizumab + tofacitinib subgroup (ROR: 4.39, 95% CI: 4.22–4.56; IC: 1.67, 95% CI: 1.55–1.79), tocilizumab + baricitinib subgroup (ROR: 4.01, 95% CI: 2.91–5.53; IC: 1.59, 95% CI: 0.53–2.53), and other-combination subgroup (ROR: 8.33, 95% CI: 7.54–9.19; IC: 2.20, 95% CI: 1.91–2.48) were significant. In general, the association with secondary infection was the highest in the combination group (ROR: 4.69, 95% CI: 4.53–4.86; IC: 1.73, 95% CI: 1.62–1.84), and was higher in the IL-6 receptor blockers group (ROR: 3.00, 95% CI: 2.95–3.05; IC: 1.29, 95% CI: 1.24–1.34) than in the JAK inhibitors group (ROR: 2.28, 95% CI: 2.25–2.31; IC: 0.99, 95% CI: 0.95–1.03). The details are shown in Table 2.

**TABLE 2:** Infection signal detection.

Drug regimen	Case number	Proportion (%)	ROR^a^			IC^b^		
			Value	Lower limit of 95% CI^b^	Higher limit of 95% CI^b^	Value	Lower limit of 95% CI^b^	Higher limit of 95% CI^b^
IL-6 receptor blockers^d^								
Tocilizumab	17,345	28.59	3.24	3.18	3.29	1.37	1.31	1.42
Sarilumab	1,481	16.92	1.64	1.55	1.73	0.61	0.43	0.79
Tocilizumab + sarilumab	29	13.55	1.26	0.85	1.86	0.29	−1.00	1.54
Total	18,855	27.07	3.00	2.95	3.05	1.29	1.24	1.34
JAK inhibitors^e^								
Baricitinib	1,127	29.85	3.42	3.19	3.67	1.43	1.21	1.65
Tofacitinib	17,161	23.85	2.53	2.49	2.57	1.11	1.05	1.16
Ruxolitinib	9,955	18.85	1.87	1.83	1.91	0.77	0.70	0.84
Baricitinib + tofacitinib	22	25.29	2.72	1.68	4.41	1.19	−0.43	2.62
Tofacitinib + ruxolitinib	3	37.5	4.82	1.15	20.16	1.76	−2.91	4.91
Baricitinib + ruxolitinib	1	100.00	-	-	-	3.17	−5.80	7.22
Total	28,269	21.97	2.28	2.25	2.31	0.99	0.95	1.03
Combination group								
Tocilizumab + tofacitinib	3 910	35.29	4.39	4.22	4.56	1.67	1.55	1.79
Tocilizumab + baricitinib	56	33.33	4.01	2.91	5.53	1.59	0.53	2.53
Tocilizumab + ruxolitinib	21	28.77	3.24	1.95	5.38	1.38	−0.32	2.84
Sarilumab + tofacitinib	33	19.08	1.89	1.30	2.77	0.78	−0.47	1.97
Sarilumab + baricitinib	1	6.67	0.57	0.08	4.36	−0.73	−5.35	4.41
Other combinations (more than three drugs)	795	50.90	8.33	7.54	9.19	2.20	1.91	2.48
Total	4 816	36.85	4.69	4.53	4.86	1.73	1.62	1.84

^a^ ROR: reporting odds ratio.

^b^ 95% CI: 95% confidence interval.

^c^ IC: information component.

^d^ IL-6, receptor blockers: interleukin-6, receptor blockers.

^e^ JAK, inhibitors: Janus kinase inhibitors.

### 3.4 Agranulocytosis signal detection

As shown in Table 3, only a few signals of drug treatment options were defined as significant, including tocilizumab (ROR: 1.61, 95% CI: 1.53–1.69; IC: 0.67, 95% CI: 0.50–0.84), tocilizumab + sarilumab (ROR: 11.92, 95% CI: 8.26–17.20; IC: 3.35, 95% CI: 1.82–4.19), ruxolitinib (ROR: 2.32, 95% CI: 2.21–2.43; IC: 1.18, 95% CI: 1.02–1.33), and tocilizumab + tofacitinib (ROR: 2.71, 95% CI: 2.47–2.98; IC: 1.40, 95% CI: 1.08–1.71). Agranulocytosis showed a higher association with the combination group (ROR: 2.36, 95% CI: 2.15–2.58; IC: 1.20, 95% CI: 0.90–1.51) than with IL-6 receptor blockers (ROR: 1.53, 95% CI: 1.45–1.60; IC: 0.59, 95% CI: 0.43–0.76) and JAK inhibitors (ROR: 1.16, 95% CI: 1.11–1.21; IC: 0.21, 95% CI: 0.07–0.35) alone.

**TABLE 3:** Agranulocytosis signal detection.

Drug regimen	Case number	Proportion (%)	ROR^a^			IC^c^		
			Value	Lower limit of 95% CI^b^	Higher limit of 95% CI^b^	Value	Lower limit of 95% CI^b^	Higher limit of 95% CI^b^
IL-6 receptor blockers^d^								
Tocilizumab	1,503	2.48	1.61	1.53	1.69	0.67	0.50	0.84
Sarilumab	104	1.19	0.76	0.63	0.92	−0.39	−1.03	0.25
Tocilizumab + sarilumab	34	15.89	11.92	8.26	17.20	3.35	1.82	4.19
Total	1,641	2.36	1.53	1.45	1.60	0.59	0.43	0.76
JAK inhibitors^e^								
Ruxolitinib	1,863	3.53	2.32	2.21	2.43	1.18	1.02	1.33
Tofacitinib	399	0.55	0.35	0.32	0.39	−1.49	−1.82	−1.16
Baricitinib	56	1.48	0.95	0.73	1.24	−0.07	−0.94	0.80
Tofacitinib + ruxolitinib	1	12.5	9.01	1.11	73.27	3.00	−4.28	5.90
Total	2,319	1.80	1.16	1.11	1.21	0.21	0.07	0.35
Combination group								
Tocilizumab + tofacitinib	456	4.12	2.71	2.47	2.98	1.40	1.08	1.71
Tocilizumab + baricitinib	6	3.57	2.34	1.03	5.28	1.19	−1.57	3.46
Tocilizumab + ruxolitinib	1	1.37	0.88	0.12	6.31	−0.19	−4.77	4.56
Sarilumab + tofacitinib	2	1.16	0.74	0.18	2.97	−0.43	−4.10	3.48
Sarilumab + baricitinib	3	20.00	15.77	4.45	55.90	3.68	−1.96	5.32
Other combination (more than three drugs)	2	0.13	0.08	0.02	0.32	−3.61	−6.84	0.68
Total	470	3.60	2.36	2.15	2.58	1.20	0.90	1.51

^a^ ROR: reporting odds ratio.

^b^ 95% CI: 95% confidence interval.

^c^ IC: information component.

^d^ IL-6, receptor blockers: interleukin-6, receptor blockers.

^e^ JAK, inhibitors: Janus kinase inhibitors.

### 3.5 Infection and agranulocytosis signals comparison in COVID-19 and non-COVID-19 patients

**FIGURE 2:**
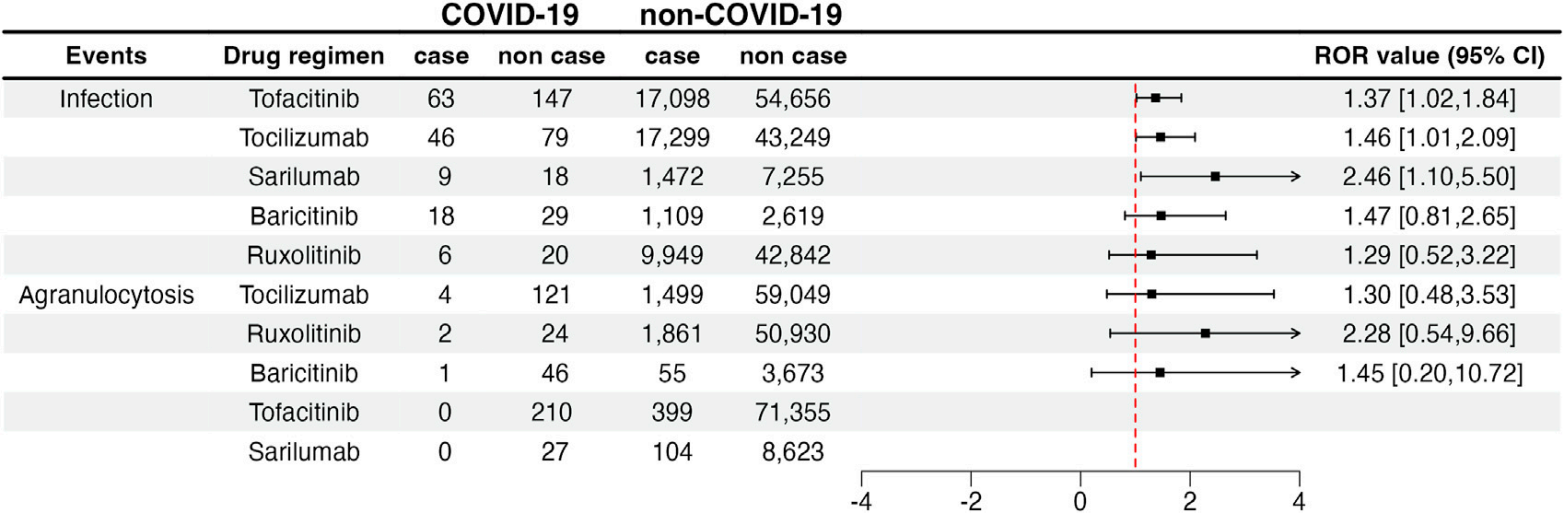
Infection and agranulocytosis signals in COVID-19 and non-COVID-19 patients. COVID-19: coronavirus disease 2019; ROR: reporting odds ratio; 95% CI: 95% confidence interval. The squares in the figure represent the ROR value, and the line segment represents the 95% CI of ROR.

We compared the reporting frequency of the target drugs between COVID-19 and non–COVID-19 patients. As for secondary infection, three drugs were reported more frequently in patients with COVID-19 than in non-COVID-19 patients. They were tofacitinib (ROR: 1.37, 95% CI: 1.02–1.84), tocilizumab (ROR: 1.46, 95% CI: 1.01–2.09), and sarilumab (ROR: 2.46, 95% CI: 1.10–5.50). Unfortunately, due to the small number of reported cases, we were not able to compare the reporting frequency of agranulocytosis between COVID-19 and non–COVID-19 patients. The details are shown in Figure 2.

## 4 Discussion

Secondary infection and agranulocytosis are life-threatening disorders that frequently occur as ADRs (Andrès et al., 2002). Drug-associated infections are usually caused by disrupted immune function, and agranulocytosis is one of the manifestations of immune dysfunction. JAK inhibitors and IL-6 receptor blockers have frequently been reported to be associated with secondary infection and agranulocytosis (Shreberk-Hassidim et al., 2017; Taylor et al., 2022). Based on our findings, secondary infection and agranulocytosis were reported more frequently in female patients. As for non-COVID-19 patients, such a result may be because females have a higher prevalence of some immune system diseases, such as rheumatoid arthritis (Myasoedova et al., 2010). However, it is not clear why the females showed a higher report probability of secondary infection and agranulocytosis among COVID-19 patients. These views are conjectures based on existing literature and cannot be proven in this study. These may need to be confirmed and explored in future research.

Signals associated with secondary infection were positive for all five target drugs. Tocilizumab and baricitinib showed the strongest relevance. Tocilizumab and baricitinib are the most used immunomodulatory therapies in COVID-19 patients (Lamontagne et al., 2020). Therefore, we recommend that patients at high risk of severe infection should be carefully evaluated before using tocilizumab and baricitinib. There were fewer reports of agranulocytosis in the FAERS database, and we calculated that tocilizumab and ruxolitinib had significant signals. It is worth noting that tocilizumab was highly correlated with both secondary infection and agranulocytosis.

Some patients may use JAK inhibitors and IL-6 receptor blockers simultaneously or sequentially in clinical practice (Lamontagne et al., 2020). Therefore, we performed signal analysis for the combined use of JAK inhibitors and IL-6 receptor blockers. Not surprisingly, the combination therapy increased the association with ADRs, for both secondary infection and agranulocytosis. Our research also demonstrated that the association of tofacitinib, tocilizumab, and sarilumab with secondary infection was higher in COVID-19 than in non–COVID-19 patients. These results allow us to speculate that the combination of JAK inhibitors and IL-6 receptor blockers has a higher association with infectious complications in patients with COVID-19. Unfortunately, due to the insufficient sample size, we were unable to compare the report frequency of secondary infection and agranulocytosis between COVID-19 and non-COVID-19 patients treated with the combination of JAK inhibitors and IL-6 receptor blockers. In addition to the target drugs, we analyzed the combination drugs in the COVID-19 group and the non–COVID-19 group. The most common combinations in both groups were methotrexate, hydroxychloroquine, and prednisone. However, we cannot rule out the influence of other combinations on the results, as the types of combinations were complex.

Our recommendations are based on the results of the study by calculating the ROR value to compare the association of different drugs with the same adverse event in real-world reports. This may provide references for clinical treatment, and the possible ADRs when using JAK inhibitors and IL-6 receptor blockers can be evaluated more carefully. However, this method cannot fully explain the severity of ADRs. Therefore, our speculations need to be further explored.

Our study first analyzed secondary infections and agranulocytosis in COVID-19 and non–COVID-19 patients treated with JAK inhibitors and IL-6 receptor blockers, and found an increased association between the combination of JAK inhibitors and IL-6 receptor blockers and ADRs. However, there are some important limitations to our study. First, the FAERS database is a spontaneous reporting system, and the potential reporting bias is hard to avoid. Second, causality cannot be inferred as the patient treatment information, including patient history and the timing of the reported medication use, is often incomplete. Hence, the mechanism of the increased association with ADRs could not be described in our study. Finally, COVID-19 patients, particularly those with severe and critical illness, are prone to secondary bacterial or fungal infections (Chong et al., 2021). However, the duration of JAK inhibitors and IL-6 receptor blockers use tends to be shorter in COVID-19 patients than in non-COVID-19 patients (Lamontagne et al., 2020). Thus, there is a bias in unpredictable directions. Therefore, more high-quality, large-sample retrospective studies or randomized controlled trials are needed to validate our findings.

## 5 Conclusion

In our study, reporting bias was unavoidable and the causation of outcomes could not be verified. Secondary infection and agranulocytosis are of concern in patients using JAK inhibitors and IL-6 receptor blockers. The combined treatment further increases the association with secondary infection and agranulocytosis. In terms of secondary infections, the association may be higher in COVID-19 than in non-COVID-19 patients. We recommend using JAK inhibitors and IL-6 receptor blockers with caution in patients at high risk of severe infection and agranulocytosis, and the combination of JAK inhibitors and IL-6 reporter blockers should be used under strict trade-offs.

## Data Availability

The original contributions presented in the study are included in the article/Supplementary Material, further inquiries can be directed to the corresponding author.
