# Supplementary material for: Agranulocytosis and secondary infection related to JAK inhibitors and IL-6 receptor blockers: a disproportionality analysis using the US Food and drug administration adverse event reporting system
Source: Front Pharmacol. 2024 Jan 9;14:1323240. doi: 10.3389/fphar.2023.1323240 (PMC10803638; doi:10.3389/fphar.2023.1323240)
Supplement: Supplementary file 1 [file Table2.DOCX]

**The Correlation between Immunoregulatory Therapy and Agranulocytosis and Infection-related Complications in Patients with COVID-19: A Disproportionality Analysis Using the US Food and Drug Administration Adverse Event Reporting System**

Chunyan Wei^1^, Wanhong Yin^2,3^, Tingting Hu^1^, Jingyi Zhang^1^, Huifang Dan^1^, Bin Wu^1,*^

**Supplementary Table S2. Identifying of target drugs with ATC codes.**

| **NO.** | **Drug name** | **ATC^a^ code** | **NO.** | **Drug name** | **ATC^a^ code** |
| --- | --- | --- | --- | --- | --- |
| 1 | Baricitinib | L04AA37 | 4 | Tocilizumab | L04AC07 |
| 2 | Tofacitinib | L04AA29 | 5 | Sarilumab | L04AC14 |
| 3 | Ruxolitinib | L01EJ01; D11AH09 |  |  |  |

^a^ACT: Anatomical Therapeutic Chemical

**Supplementary Table S3. Algorithm for disproportionate analyses**

| **Drugs** | **target^a^ event cases** | **All other adverse event cases** |
| --- | --- | --- |
| Target drug | a | b |
| All other drugs | c | d |
| ROR^a^ = $\frac{a/b}{\text{c}/d}$  95%CI^b^ for ROR^a^ = $e^{\text{ln(}\text{R}\text{OR)}\pm\text{1.96}\sqrt{(\frac{1}{a}+\frac{1}{b}+\frac{1}{c}+\frac{1}{d})}}$ | | |
| IC^c^ = log_2_$\frac{a(a+b+c+d)}{\text{(a+b)(a}+c)}$  E(IC^c^) = log_2_$\frac{(a+\gamma11)(N+\alpha)(N+\beta)}{\text{(N+γ)(a}+b+\alpha1)(a+c+\beta1)}$  V(IC^c^) ≈ ($\frac{1}{\text{log2}}$)^2^[$\frac{N-a+\gamma-\gamma11)}{\text{(a+γ11)(1}+N+\gamma)}+\frac{N-a-b+\alpha-\alpha1)}{\text{(a+b+α1)(1}+N+\alpha)}+\frac{N-a-c+\beta-\beta1)}{\text{(a+c+β1)(1}+N+\beta)}$]  γ = γ_11_$\frac{(N+\alpha)(N+\beta)}{\text{(a+b+α1)(a}+c+\beta1)}$  95%CI^b^ for IC^c^ = E(IC^c^) ± 1.96$\sqrt{V(IC)}$  Where α=α_1_+α_2_, β=β_1_+β_2_, N=a+b+c+d, and the value of α_1_, α_2_, β_1_, β_2_ and γ_11_ were defined as 1. | | |

^a^ROR: reporting odds ratio; ^b^CI: confidence interval. ^c^IC: information component.
